# Improving the Berry Quality and Antioxidant Potential of Flame Seedless Grapes by Foliar Application of Chitosan–Phenylalanine Nanocomposites (CS–Phe NCs)

**DOI:** 10.3390/nano11092287

**Published:** 2021-09-02

**Authors:** Gholamreza Gohari, Elnaz Zareei, Muhittin Kulak, Parisa Labib, Roghayeh Mahmoudi, Sima Panahirad, Hessam Jafari, Gholamreza Mahdavinia, Antonio Juárez-Maldonado, José M. Lorenzo

**Affiliations:** 1 Department of Horticultural Sciences, Faculty of Agriculture, University of Maragheh, Maragheh 55181-83111, Iran; 2 Department of Horticultural Science, Faculty of Agriculture, University of Kurdistan, Sanandaj 66177-10175, Iran; elnaz.zareei09@gmail.com; 3 Department of Herbal and Animal Production, Vocational School of Technical Sciences, Igdir University, Igdir 18900, Turkey; muhyttynx@gmail.com; 4 Polymer Research Laboratory, Department of Chemistry, Faculty of Science, University of Maragheh, Maragheh 55181-83111, Iran; p.labib@stu.maragheh.ac.ir (P.L.); gholamreza.mahdavinia@gmail.com (G.M.); 5 Department of Horticulture, Faculty of Agriculture, University of Zanjan, Zanjan 45371-38791, Iran; r.mahmoudie69@gmail.com; 6 Department of Horticultural Sciences, Faculty of Agriculture, University of Tabriz, Tabriz 51666-16471, Iran; s.panahirad@tabrizu.ac.ir; 7 Department of Organic Chemistry, Faculty of Chemistry, University of Tabriz, Tabriz 51666-16471, Iran; h.jafari@tabriz.ac.ir; 8 Departamento de Botánica, Universidad Autónoma Agraria Antonio Narro, Saltillo 25315, Mexico; 9 Centro Tecnológico de la Carne de Galicia, Rúa Galicia N° 4, Parque Tecnológico de Galicia, San Cibrao das Viñas, 32900 Ourense, Spain; 10 Área de Tecnología de los Alimentos, Facultad de Ciencias de Ourense, Universidad de Vigo, 32004 Ourense, Spain

**Keywords:** edible coating, non-enzymatic antioxidants, phenylalanine ammonia-lyase, shelf life, *Vitis vinifera*

## Abstract

The production and sustainability of grape berries with high quality and health-promoting properties is a major goal. In this regard, nano-engineered materials are being used for improving the quality and marketability of berries. In this study, we investigated the potential role of chitosan–phenylalanine nanocomposites (CS–Phe NCs) in improving the quality of Flame Seedless (*Vitis vinifera* L.) grape berries, such as titratable acidity (TA), pH, total soluble solids (TSS), ascorbic acid, total phenolics, total flavonoids, anthocyanin, 2,2-diphenyl-1-picryl-hydrazyl-hydrate (DPPH) radical scavenging activity, and phenylalanine ammonia-lyase (PAL) activity. In this context, grape berries collected in two growing seasons (2018–2019) were screened. Regarding the experimental design, the treatments included chitosan at a 0.5% concentration (CS 0.5%), phenylalanine at 5 mM and 10 mM concentrations (Phe 5 mM and Phe 10 mM), and chitosan–phenylalanine nanocomposites (CS–Phe NCs) at 5 mM and 10 mM concentrations. The lowest TA was recorded in grape berries treated with CS–Phe NCs with a 10 mM concentration. However, treatments enhanced with TSS, which reached the highest value with 10 mM of CS–Phe NCs, were reflected as the highest ratio of TSS/TA with 10 mM of CS–Phe NC treatment. Nanocomposites (NCs) also increased pH values in both study years compared to the control. Similarly, the ascorbic acid and total phenolic content increased in response to NP treatment, reaching the highest value with 5 mM and 10 mM of CS–Phe NCs in 2018 and 2019, respectively. The highest flavonoid content was observed with 5 mM of CS–Phe NCs in both study years. In addition, the anthocyanin content increased with 5 and 10 mM of CS–Phe NCs. PAL activity was found to be the highest with 5 mM of CS–Phe NCs in both study years. In addition, in accordance with the increase in PAL activity, increased total phenolics and anthocyanin, and higher DPPH radical scavenging activity of the grapes were recorded with the treatments compared to the control. As deduced from the findings, the coating substantially influenced the metabolic pathway, and the subsequent alterations induced by the treatments were notably appreciated due to there being no adverse impacts perceived.

## 1. Introduction

Grapevine is a major fruit crop cultivated in all continents and consumed in various forms, such as fresh fruit and other processed products (e.g., vines and juice). It is one of the most economically significant sources for the food industry due to the soluble sugars, phenolics, flavonoids, organic acids, and aromatic compounds available [1]. To maintain the high quality of grape berries, especially under changing environmental conditions, chitosan nanomaterials have recently been used [2].

After cellulose, chitosan (β-(1,4)-2-amino-2-deoxy-D-glucose) is the second most abundant polysaccharide deacetylated derivative of chitin in nature [3]. Due to the existence of functional groups, especially amino groups, chitosan plays an important role in the interaction with negatively charged molecules because of the protonation phenomenon [4]. As an environment-friendly polymer, chitosan is used as a coating for fruits during handling and packaging as protection against dehydration and microbial activity [5]. Previous studies have reported that chitosan, combined with other chemicals, has a positive impact on the control of foliar diseases in some crops [6].

Nanoscale particles (1–100 nm) are widely used in industry, medicine, and agriculture [7,8]. These small particles have significant impacts on plant growth and tolerance to biotic and abiotic stresses and, additionally, on fruit quality [9,10,11]. In the past decade, chitosan-based nanomaterials have been widely used in various forms, including nanoparticles, microfibers, and microbeads, in numerous agriculture applications [12]. One of the reasons for this is the availability of functional groups in the chemical structure of chitosan, enabling it to react effortlessly with other active compounds. Being deemed an elicitor or carrier for plant growth regulators, agricultural applications of chitosan are common and reported for many crops [13].

Considering chitosan coatings, chitosan-g-salicylic acid coating decreased weight loss and respiration rate, preserved higher total soluble solids and chlorophyll content, and limited the increase in malondialdehyde (MDA) content and electrolyte leakage in cucumber plants stored at 2 °C for 12 days plus 20 °C for 2 days [14]. Enhancement of the storage ability of grape plants treated with chitosan and polyvinyl alcohol (CS–PVA) biopolymer coating blending with salicylic acid (SA), through increasing antioxidant activity, has been reported [15]. CS–PVA blending with SA has been suggested to protect phenolic substances, such as total phenol and flavonoids, due to the inhibited activity of polyphenol oxidase [15]. Chitosan-based melatonin layer-by-layer assembly with 1.2% (*w*/*v*) chitosan, 0.8% (*w*/*v*) carboxymethyl cellulose, and 50 mg L^−1^ of melatonin treatment delays chlorophyll degradation and maintains the shelf life of fresh products [16]. Nasr et al. [17] reported that postharvest application of phenylalanine-coated chitosan nanoparticles can decrease storage disorder during cold storage of persimmon fruits. Sheikhalipour et al. [18] reported that foliar application of chitosan-based selenium nanocomposites can increase plant tolerance to abiotic stress and also fruit quality in *Momordica charantia* and *Stevia rebaudiana* Bertoni.

Phenylalanine is one of the significant compounds that might provide potential results in order to improve grape quality because its nitrogen sources, being a substrate for phenylalanine ammonia-lyase (PAL)-catalyzing reactions regarding the conversion of phenylalanine into cinnamic acid, are a first step in the biosynthesis of phenolic compounds. It has been reported that foliar application of phenylalanine improves the phenolic and grape amino acid composition of grapevines, in addition to their carotenoid and nitrogen content [19].

Following on from and expanding the available research with parallel ideas and considering phenylalanine as a potential active compound, the aim of this study was to develop an innovative and active chitosan-based coating with phenylalanine and apply these newly engineered chitosan-based nanoparticles to grape berries to increase their quality during preharvest treatment.

## 2. Materials and Methods

### 2.1. Chitosan–Phenylalanine Nanocomposite (CS–Phe NC) Synthesis

To prepare CS–Phe NCs, a biopolymer was used to load Phe. In this study, 0.1 g of low-molecular-weight nanocomposite (CS) powder was added to 25 mL of 1 wt% acetic acid solution and stirred for 2 h at 70 °C using a magnetic stirrer to obtain a clear CS solution. The prepared Phe solution (0.1 g in 15 mL of distilled water) was added to the CS solution, which was then rapidly stirred for 1 h. Sodium tripolyphosphate (TPP) was used as a cross-linker with a ratio of 2.5 to the CS content. Briefly, 0.04 g of TPP was dissolved in 5 mL of distilled water and then slowly added to the CS–Phe solution. TPP in cross-linking with CS nanoparticles causes coagulation, and this mixture coagulant was stirred overnight and then rinsed several times with distilled water to remove unreacted material from the supernatant.

### 2.2. Chitosan–Phenylalanine Nanocomposite (CS–Phe NC) Characterization

After the sample dried, the general techniques of scanning electron microscopy (SEM) and energy dispersive spectroscopy (EDS) (VEGAII, XMU, Brno, Czech Republic), transmission electron microscopy (TEM) (Philips CM10 operating at 60 kV tension, Eindhoven, The Netherlands ), fourier transform infrared spectroscopy (FT-IR) (Bruker 113 V FT-IR spectrometer, Ettlingen, Germany ), and X-ray diffraction (XRD) (Siemens D-500 X-ray diffractometer, Karlsruhe, Germany) were performed.

### 2.3. Experimental Site and Fruit Treatments

The experiment was conducted at a vineyard in Maragheh Province, Iran (longitude 46°53′ E, latitude 37°38′ N). Seven-year-old grapevines (*Vitis vinifera* cv. Flame Seedless) with a relatively similar growth state were exposed to different concentrations of chitosan alone and in combination with phenylalanine for 2 years (2018 and 2019). The experiment was performed using a completely randomized design (CRD) in three replications. The treatments included chitosan at 0.5% concentration (CS 0.5%), phenylalanine at 5 mM and 10 mM concentration (Phe 5 mM and Phe 10 mM), and chitosan–phenylalanine nanocomposites (Cs–Phe NCs) at 5 mM and 10 mM concentration.

Each experimental group included two plants that corresponded to three clusters with the same size, maturity, and development. For the treatments, the entire clusters of grape berries were sprayed with nanocomposites along with the surfactant Tween^®^ 20 twice at 1 week intervals in the early morning. Spraying at 1 week intervals was carried out to ensure that all clusters received sufficient amounts of treatment pre-veraison in the case of any rain, deficiency, or late development of some berries. Similarly, the same amount of distilled water with Tween^®^ 20 was applied to untreated plants. For sampling, most of the berries were similar in color, softness, and development level. In addition, the same spraying procedure was performed for both study years.

### 2.4. Quality Assessment

#### 2.4.1. Titratable Acidity (TA) and pH

The titratable acidity (TA) was determined by titration with 0.1 N sodium hydroxide (NaOH) solution with up to 8.2 pH. The TA was expressed as g tartaric acid L^−1^ FW. The pH of samples was determined using a pH meter (Mi150, Milwaukee, Szeged, Hungary) [20].

#### 2.4.2. Total Soluble Solids (TSS)

Total soluble solids (TSS) were measured at 20 °C using a refractometer (PAL-1, Atago, Japan). The TSS was expressed as a percentage [21].

#### 2.4.3. Ascorbic Acid Content

The ascorbic acid content was assessed using titration with 2,6-dichlorophenolindophenol (DCPIP) reagent, as described by De Bolt et al. [22]. Briefly, 10 mL of 3% metaphosphoric acid extract was titrated with 4% DCPIP solution up to 100 mL for 10 to 15 s. The ascorbic acid content was expressed as mg ascorbic acid equivalent 100 g^−1^ fresh weight (FW).

### 2.5. Total Phenolic Content

After harvest, whole berries were snipped from clusters. Freeze-dried berry samples were subjected to extraction with MeOH/H_2_O/acetic acid (70:29:1, *v*/*v*/*v*) with a 4/1 (*v*/*w*) solvent/sample ratio. Shaker-assisted extraction was performed at 300 rpm for 2 h at room temperature. Then, the supernatant was removed, followed by resuspending the pellet with 4 mL of the solvent. A second shaker-assisted extraction was used under the conditions of the first extraction. The final mixture was combined and centrifuged at 10,000× *g* for 10 min. After centrifugation, the supernatant was collected and preserved at −20 °C for further analysis. The extractions were repeated three times [23].

The total phenolic content (TPC) was quantified using Folin–Ciocalteu reagent based on Singleton and Rossi assay [24]. Briefly, 2.5 mL of Folin–Ciocalteu reagent and 450 µL of deionized water were added to a test tube containing 50 µL of methanolic grape extract. After 10 min, 2 mL of 7.5% sodium carbonate was added to the mixture. Subsequently, the mixture was kept in the dark at room temperature for 90 min. Absorbance was recorded at 755 nm using a Spekol 1500 UV–VIS spectrophotometer (Analytik Jena, Jena, Germany). The total phenolic content was expressed as mg gallic acid 100 g^−1^ FW.

### 2.6. Total Flavonoid Content

The total flavonoid content was determined colorimetrically using aluminum chloride (AlCl_3_) solution [25]. For this purpose, methanolic grape extract was taken in 75 µL of 5% sodium nitrite (NaNO_2_), 0.15 mL of 10% aluminum chloride, and 0.5 mL of 1 M sodium hydroxide (NaOH). Then, the mixture was diluted to 2.5 mL with ddH_2_O. The absorbance of the samples was recorded at 510 nm, and the results were expressed as mg Quercetin equivalent 100 g^−1^ FW.

### 2.7. Anthocyanin Content

Grape berries were extracted in 2% HCl in methanol for 24 h in the dark at room temperature. The total anthocyanin content was measured based on the difference in pH [26]. Fruit extracts were diluted to an appropriate concentration with potassium chloride buffer (pH 1.0). The spectrophotometer was blanked with distilled water. Two dilutions of each sample were prepared, one with potassium chloride buffer (pH 1.0) and the other with sodium acetate buffer (pH 4.5), and the dilutions were allowed to stand for 15 min. Absorbance was recorded at 520 and 700 nm using a Spekol 1500 UV–VIS spectrophotometer (Analytik Jena, Jena, Germany). The absorbance of the sample was calculated using the following formula

Absorbance = (A520 − A700) pH 1.0 − (A520 − A700) pH 4.5

The concentration of total monomeric anthocyanins was calculated using the following formula

Total anthocyanin content = (Ab/εL) × 1000 × MW × DF

where L is the path length of the cell, MW = 528.89 gmol^−1^, and ε = 28,000 L cm^−1^ mol^−1^. The total anthocyanin content was expressed in mg oenin (the main anthocyanin from grapes) 100 g^−1^ FW.

### 2.8. Scavenging 2,2-Diphenyl-1-picrylhydrazyl (DPPH) Free Radical Activity

To determine DPPH radical scavenging activity, 1 g of grape berries was extracted with 0.1% HCl acidified with 80% methanol (8 mL *v*/*v*) and then centrifuged at 10,000× *g* for 20 min at 4 °C. The antioxidant capacity of samples was spectrophotometrically measured using the 2,2-diphenyl-1-picrylhydrazyl (DPPH) free radical assay, as defined by Brand-Williams et al. [27]. Absorbance was measured at 515 nm using a Spekol 1500 UV–VIS spectrophotometer (Analytik Jena, Jena, Germany) at different time intervals until the reaction reached a plateau. Antioxidant capacity was expressed as the percentage inhibition of the DPPH radical, and the DPPH radical scavenging activity (RSA) was calculated as follows

% Radical scavenging activity (RSA) = (Ablank − Asamp)/(Ablank) × 100

### 2.9. PAL Enzyme Activity

The activity of phenylalanine ammonia-lyase (PAL) was measured according to Zucker [28] and Oraei et al. [23]. The assay mixture contained 50 mM Tris−HCl buffer (pH 8.8), 10 mM L-phenylalanine, and 0.1 mL of enzyme extract. Briefly, the rate of conversion of L-phenylalanine to trans-cinnamic acid was determined at 290 nm for 5 min using a Spekol 1500 UV–VIS spectrophotometer (Analytik Jena, Jena, Germany).

### 2.10. Statistical Analysis

All obtained data were analyzed using SAS software ver. 9.1 (SAS Institute Inc., Cary, NC, USA) and analysis of variance (ANOVA). Differences between means were compared using Duncan’s multiple-range test at a 95% level of probability.

## 3. Results and Discussion

### 3.1. Characterization of the Synthesized Chitosan–Phenylalanine Nanocomposites (CS–Phe NCs)

**Figure 1:**
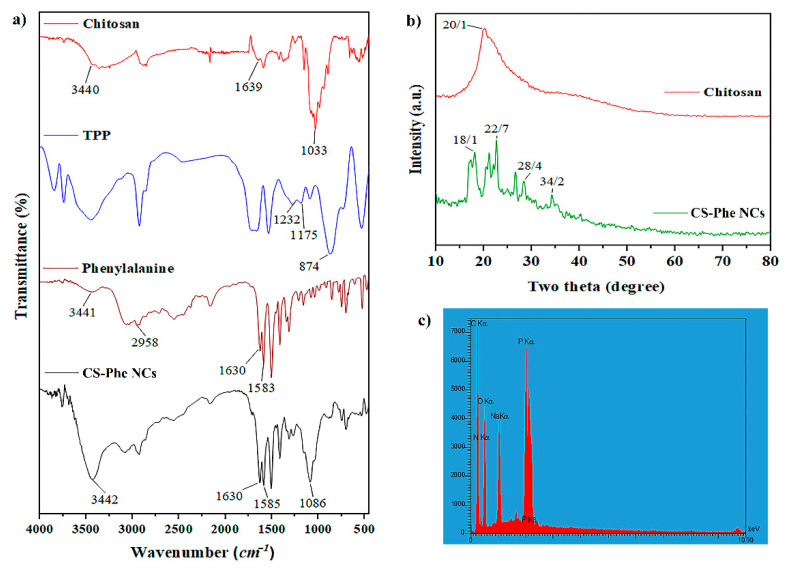
FT-IR (**a**), XRD (**b**), and EDS (**c**) analyses of raw materials and chitosan–phenylalanine nanocomposites (CS–Phe NCs).

FT-IR analysis was performed to identify the chemical structure and functional groups of raw materials and the prepared samples. The results showed that Phe was loaded on the CS–TPP nanocarrier (Figure 1a). Based on the CS spectrum, the peaks observed at 3440, 1639, and 1033 cm^−1^ displayed the stretching vibrations of –OH, –NH_2_, and C–O–C functional groups, respectively [29]. Regarding the Phe spectrum, the peak observed at 1583 cm^−1^ showed the stretching vibrations of the C=O group. The existence of –COOH, C–H, and C=C groups in the Phe compound was confirmed by peaks at 3441, 2958, and 1630 cm^−1^, respectively [30]. According to the TPP spectrum, the peaks observed at 1232, 1174, and 874 cm^−1^ confirmed the stretching vibrations of P–O, P=O, and P–O–P bonds, respectively [31]. In the prepared nanocarrier spectrum, the peak at 1630 cm^−1^ was related to the bending vibrations of –NH_2_, which showed displacement from 1657 cm^−1^ in the main peak of the CS spectrum. A new peak also appeared at 1585 cm^−1^, which corresponded to the cross-linking between the TPP and –NH_3_^+^ groups in CS, confirming the loading of Phe on the CS–TPP nanocarrier.

X-ray diffraction analysis was performed to identify the compounds and phases in the structure of materials. In the chitosan XRD pattern, the wide peak observed at 2θ = 20.1° indicated the quasi-crystalline nature of CS [30]. In the CS–Phe nanocarrier pattern, several peaks appeared at 2θ = 18.1°, 22.7°, 28.4°, and 34.2°, which confirmed the dense network of CS polymer chains cross-linked by TPP (Figure 1b). Considering EDS analysis (Figure 1c), peaks related to P, C, O, and N were observed in the spectra. According to the structure of CS, the peaks of C, N, and O show the presence of CS, and the peaks of P and Na show the interaction between CS and TPP in the nanocarrier.

**Figure 2:**
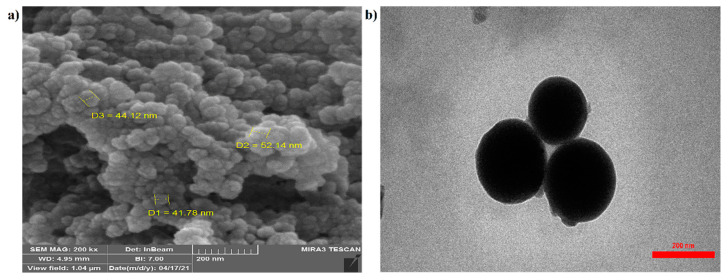
SEM (**a**) and TEM (**b**) analyses of raw materials and chitosan–phenylalanine nanocomposites (CS–Phe NCs).

The scanning electron microscope (SEM) image shown in (Figure 2a) reveals the surface morphology of the prepared spherical nanocarriers. The structure had no porosity, the particle shape was good, and the particle size was homogeneous. The transmission electron microscope (TEM) image (Figure 2b) explains the internal structure of the material, which in the nanocarrier image was measured to be about 150 nm in size.

### 3.2. Titratable Acidity (TA), Total Soluble Solids (TSS), and TSS/TA

**Figure 3:**
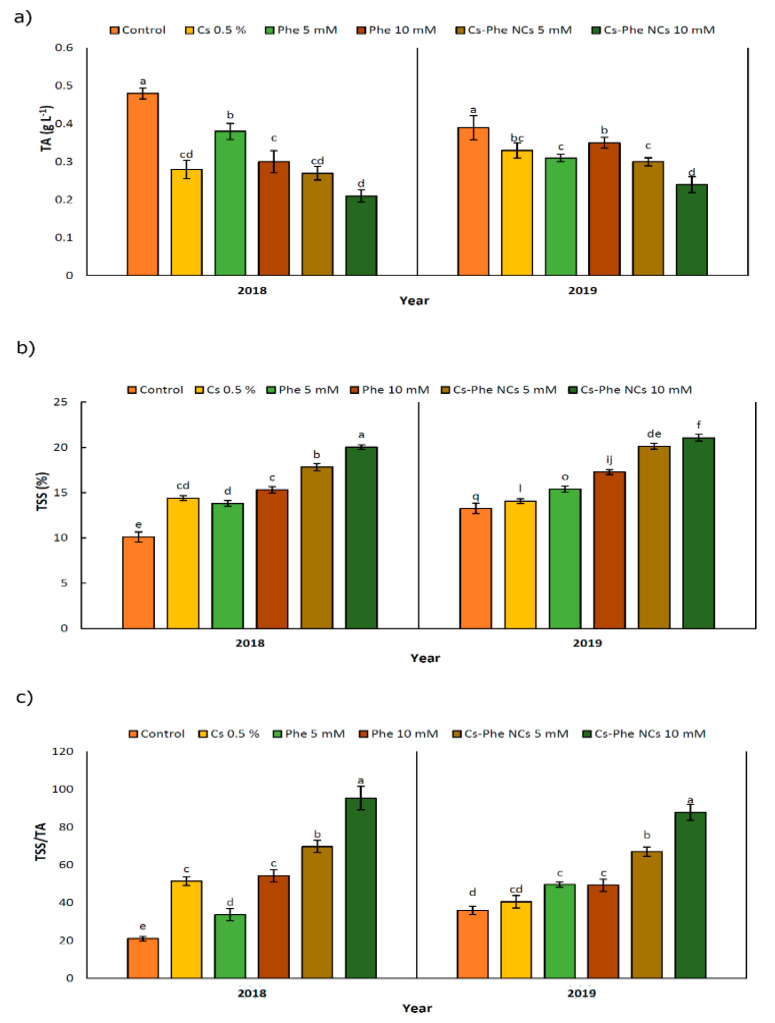
Effects of 0 (control), Cs (0.5%), Phe (5 and 10 mM), and CS–Phe NCs (5 and 10 mM) on the TA (**a**), TSS (**b**), and TSS/TA (**c**) of *V. vinifera* cv. Flame Seedless berries. The SDs denoted by lowercase letters in the same treatment indicate significant differences at *p* < 0.05 according to Duncan’s test.

Chitosan–phenylalanine NPs substantially affected the values of TA, TSS, and TSS/TA in 2 years (Figure 3). Specifically, TA declined after NP treatments compared to the control in 2 years. In 2018 and 2019, the lowest TA was observed in plants treated with CS–Phe NCs at a 10 mM concentration (Figure 3a). However, TSS increased after treatments, reaching the highest value with the 10 mM CS–Phe NC treatment in both study years (Figure 3b), being reflected as the highest ratio of TSS/TA with the 10 mM CS–Phe NC treatment (Figure 3c). TA and TSS, as well as pH, are important factors in flavor indexes and fruit quality, suggesting dominant organic acid concentration in fruits [32].

In this study, notable decreases of 56.25% and 47.92% in TA were recorded with 10 mM of CS–Phe NCs compared to the control in 2018 and 2019, respectively. As reported by Ishkeh et al. [33], total acidity is an indicator of the senescence status of fruits and a relevant decline in the TA might be a consequence of higher respiration and ripening rates, which are related to the consumption and conversion of organic acids to sugars during respiration [33].

Considering TSS, 10 mM of CS–Phe NC increased TSS by 100.99% and 58.90% in 2018 and 2019, respectively, compared to the control, suggesting that activity of the cell wall degrading enzymes increases, which is then reflected in significant increases in TSS [34]. The current findings could reveal that relevant CS–Phe NC treatments may delay aging and prevent the possible decline in TSS, as in the case of Cu–chitosan NPs and the TA and TSS in tomatoes during storage [35]. Zhu et al. [36] showed that the nano-SiO_2_–chitosan complex increases the TA and TSS in tomatoes at the end of storage compared to the control.

### 3.3. pH

**Figure 4:**
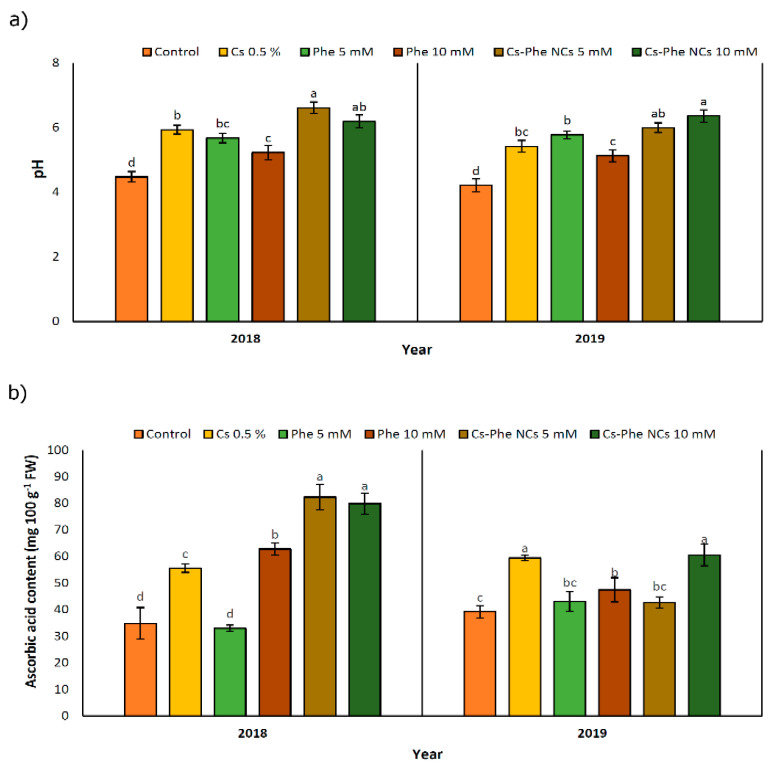
Effect of 0 (control), CS (0.5%), Phe (5 and 10 mM), and CS–Phe NCs (5 and 10 mM) on pH (**a**) and ascorbic acid content (**b**) of *V. vinifera* cv. Flame Seedless berries. The SDs denoted by lowercase letters in the same treatment indicate significant differences at *p* < 0.05 according to Duncan’s test.

In both study years, NP treatments increased pH compared to the control, and the highest value was observed at 10 mM of CS–Phe NCs followed by 5 mM of CS–Phe NCs in 2019, but at 5 mM of CS–Phe NCs followed by 10 mM of CS–Phe NCs in 2018 (Figure 4a). Being coupled with the decline in TA, pH increased, which was attributed to the consumption of organic acids to provide the required energy for respiration during storage [37].

### 3.4. Ascorbic Acid (Vitamin C)

NP treatments led to an increase in the ascorbic acid content in both study years. In 2018 and 2019, 5 mM and 10 mM of CS–Phe NCs resulted in the highest ascorbic acid content, respectively (Figure 4b). However, in 2018, Phe treatment at 5 mM concentration decreased the ascorbic acid content. Except for 10 mM of CS–Phe NCs and 0.5% CS, the remaining NP treatments had no significant differences compared to the control group. The relevant increases can be attributed to low respiration rates, as reported in many crops [38]. Interestingly, no clear findings concerned with coatings with or without phenylalanine were noted for ascorbic acid, revealing the necessity of studies on the possible impacts of chitosan and its conjugation with phenylalanine on oxygen and carbon dioxide concentrations related to respiration.

Ascorbic acid is a widely investigated non-enzymatic antioxidant playing a significant role in free radical scavenging [39]. CS–Phe NCs treatment at 5 mM concentration increased the ascorbic acid content by about 136.40% and 9.13% in 2018 and 2019, respectively, compared to the control. A similar improvement was noted with the chitosan and phytic acid combination in fresh-cut lotus [40].

### 3.5. Total Phenolic Content

**Figure 5:**
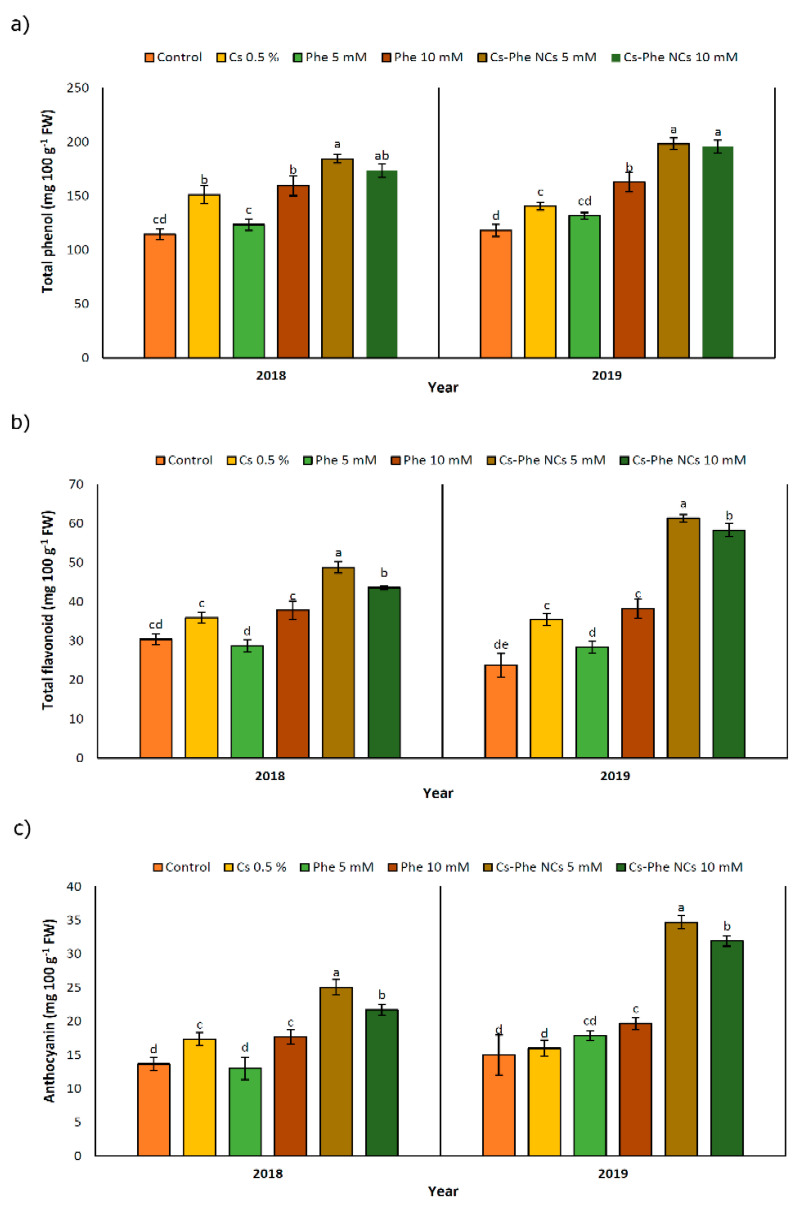
Effect of 0 (control), CS (0.5%), Phe (5 and 10 mM), and CS–Phe NCs (5 and 10 mM) on total phenol (**a**), total flavonoid (**b**), and anthocyanin (**c**) content of *V. vinifera* cv. Flame Seedless berries. The SDs denoted by lowercase letters in the same treatment indicate significant differences at *p* < 0.05 according to Duncan’s test.

Being confined to the plant kingdom, phenolic compounds are important secondary metabolites due to their diverse chemical structures. Corresponding to their diversity, relevant compounds confer a certain degree of protective roles in response to biotic and abiotic stress factors by acting as non-enzymatic antioxidants [39]. As reported before [33], phenolic compounds with their natural fungicide potential might indirectly decrease the decay extension for horticultural crops, in particular. The well-known phenolic compound biosynthesis pathway is based on phenylalanine, tyrosine, and tryptophan amino acids [41]. Along with the target and hypothesis of yielding a higher phenolic compound content, chitosan nanoparticles were coated with phenylalanine as a significant precursor of phenolic compounds. Accordingly, the total phenolic content was significantly affected with NC treatments, reaching the highest value at 5 mM and 10 mM of CS–Phe NCs in 2018 and 2019, respectively (Figure 5a). In particular, chitosan coated with phenylalanine led to a greater improvement in the phenolic composition of grape berries compared to the non-treated groups. However, the other chitosan and phenylalanine treatments also significantly enhanced the production of phenolic compounds. Interestingly, this situation resembles the case of salicylic acid-pre-treated pea seeds containing augmented endogenous levels of salicylic acid, which were revealed to be the product of de novo synthesis, not originating from the exogenously given salicylic acid that was taken up and mobilized by the plants [42]. Similar increases in the production of raspberries treated by chitosan nano-emulsion have been reported [33].

### 3.6. Total Flavonoid Content

The flavonoid content was affected by different NC treatments. In both study years, plants treated with 5 mM of Cs–Phe NCs presented the highest flavonoid content (Figure 5b). It is noticeable that the rate of enhancement with regard to the flavonoid content was higher in 2019 than in 2018. Being referred to as specialized metabolites widely distributed in horticultural crops, significant health-promoting roles have been attributed to flavonoids acting as free radical scavengers and, subsequently, protecting plants from oxidative damage [43]. As seen in Figure 5b, both concentrations of CS–Phe NCs increased the flavonoid content in both study years. The relevant findings are consistent with Ishkeh et al. [33], who reported that raspberries treated with a chitosan nano-emulsion, especially at 5 g L^−1^, exhibited a higher flavonoid content. The higher levels of flavonoids compared to the non-treated groups might be attributed to a reduced conversion of flavonoids to other secondary metabolites or an enhanced oxygen barrier ability playing a significant role in retarding the decline in the flavonoid content [44]. These findings suggest the requirement of deeper research on the bio-conversion of metabolites as a response to chitosan and its conjugation.

### 3.7. Anthocyanin

Based on the results, NC treatments led to an increase in anthocyanins. CS–Phe NCs at both concentrations (5 and 10 mM) showed the highest amount of anthocyanin accumulation in 2018 and 2019 (Figure 5c); however, in 2019, the rate of accumulation was higher. As in the case of phenolic compounds and flavonoids, research suggests that the anthocyanin content in plants is upregulated under biotic and abiotic stresses [43]. The increase in content is associated with the high capacity of anthocyanins to eliminate free radicals or act as hydrogen suppliers [45]. However, as in the case of horticultural crops, the prominent functions of anthocyanins are associated with their role in fruit color and quality [46]. In this regard, keeping the anthocyanin content at a desired level is the main target for a high quality of crops. Therefore, we investigated the potential effects of CS–Phe NC treatments on improving the quality of grape berries. CS–Phe NC treatment led to a higher concentration of anthocyanins in grape berries compared to the control. Our results are in line with the findings of studies on chitosan nano-emulsion-treated fruits [33].

### 3.8. Scavenging 2,2-Diphenyl-1-picrylhydrazyl (DPPH) Free Radical Activity

**Figure 6:**
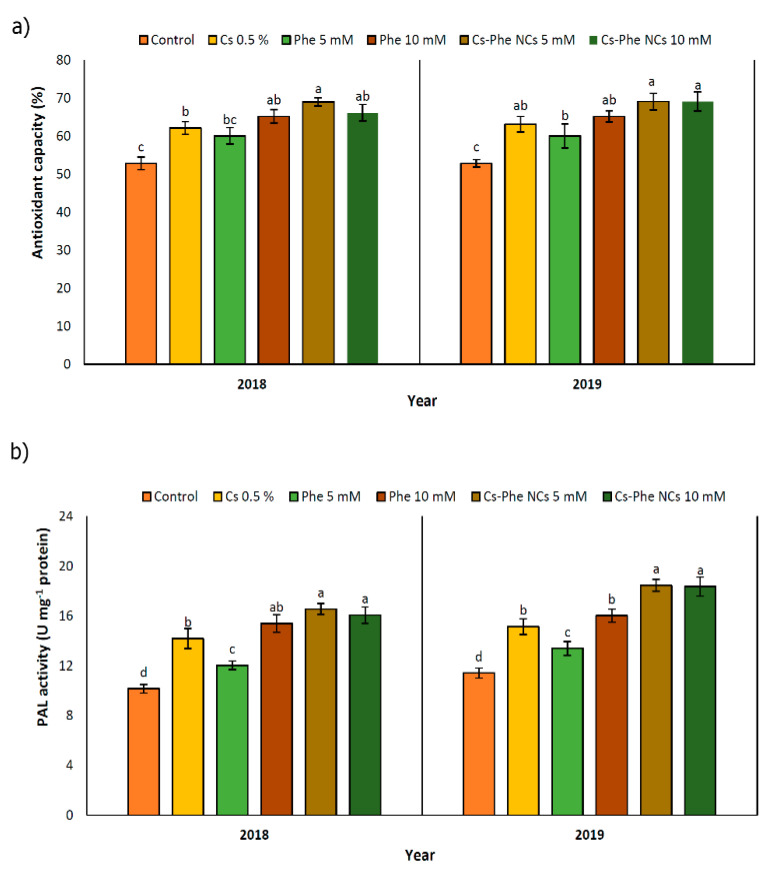
Effect of 0 (control), CS (0.5%), Phe (5 and 10 mM), and CS–Phe NCs (5 and 10 mM) on antioxidant capacity (**a**) and PAL activity (**b**) of *V. vinifera* cv. Flame Seedless berries. The SDs denoted by lowercase letters in the same treatment indicate significant differences at *p* < 0.05 according to Duncan’s test.

The highest antioxidant capacity was detected with 5 mM of CS–Phe NCs in both 2018 and 2019. Furthermore, in 2019, there was no significant difference between the 5 and 10 mM CS–Phe NC treatments (Figure 6a). Free radicals are common to all organisms and are by-products of metabolic reactions. However, their levels are regulated by a system involving enzymatic and non-enzymatic antioxidants. As is well known, phenolics, flavonoids, and anthocyanins are units of the non-enzymatic antioxidant defense system [39,47]. Along with enhanced phenolic, flavonoid, and anthocyanin content, the DPPH radical scavenging property of grape berries also increased with all NP treatments compared to the control in both study years (Figure 5a). The use of edible coatings increases the capacity of the fruit antioxidant system and protects cells against oxidative stress and pathogen attack [33].

### 3.9. PAL Activity

PAL activity was enhanced with all NP treatments in both study years. Compared to the control, 5 mM of CS–Phe NCs showed the maximum PAL activity in grape berries in both study years (Figure 6b). PAL plays a key role in different phenolic biosynthesis pathways, such as the phenylpropanoid pathway [48]. PAL activity decreases during the maturity and postharvest stages [49]. In addition, with the increase in polyphenol oxidase (PPO) activity during senescence, the consumption of polyphenols increases and, as a result, the content of total phenolic compounds decreases with the aging of fruit tissue [50]. The results of our study exhibited that CS–Phe NC treatments enhanced PAL activity, resulting in higher phenolic content. Due to the significant role of phenolics in both plant health and, subsequently, human health, reducing phenolic oxidation is of great interest, and, in this regard, edible coatings with chitosan can be used to protect the surface of products [51]. Significantly, chitosan acts as an elicitor in the activation of PAL and other enzymes playing a role in polyphenol biosynthesis [52]. Increased PAL activity has also been reported in plants exposed to 5 g L^−1^ of chitosan nano-emulsion on the ninth day after storage.

## 4. Conclusions

Chitosan coatings, with and without phenylalanine, significantly affect the metabolic pathways, but chitosan coatings with phenylalanine are notably more effective with regard to the investigated parameters of grape berries. As a novel and interesting finding, we observed that chitosan coated with phenylalanine as a functional compound triggered the activity of PAL, a significant enzyme in the biosynthesis pathway of phenolics. In accordance with PAL induction, the responses were positively manifested as higher phenolic, flavonoid, and anthocyanin content in grape berries. As reported by many researchers, the higher levels of relevant metabolites are considered elicitors of higher DPPH radical scavenging activity. Moreover, titratable acidity significantly reduces with 10 mM of CS–Phe NCs, while total soluble solids increase with 10 mM of CS–Phe NCs. The present findings suggest that functionalized compounds applied via coating may act as anti-stress and antioxidant agents by detoxifying the ROS in fruits, enhancing postharvest life and quality.

## Data Availability

The data that support the findings of this study are available from the corresponding author upon reasonable request.
